# Galvanotactic Migration of Glioblastoma and Brain Metastases Cells

**DOI:** 10.3390/life12040580

**Published:** 2022-04-14

**Authors:** Falko Lange, Jakob Venus, Daria Shams Esfand Abady, Katrin Porath, Anne Einsle, Tina Sellmann, Valentin Neubert, Gesine Reichart, Michael Linnebacher, Rüdiger Köhling, Timo Kirschstein

**Affiliations:** 1 Oscar-Langendorff-Institute of Physiology, Rostock University Medical Center, 18057 Rostock, Germany; jakob.venus@uni-rostock.de (J.V.); daria.abady@uni-rostock.de (D.S.E.A.); katrin.porath@uni-rostock.de (K.P.); anne.einsle@med.uni-rostock.de (A.E.); tina.sellmann@uni-rostock.de (T.S.); valentin.neubert@uni-rostock.de (V.N.); gesine.reichart@uni-rostock.de (G.R.); ruediger.koehling@uni-rostock.de (R.K.); timo.kirschstein@uni-rostock.de (T.K.); 2 Center for Transdisciplinary Neurosciences Rostock, University of Rostock, 18147 Rostock, Germany; 3 Molecular Oncology and Immunotherapy, Rostock University Medical Center, 18057 Rostock, Germany; michael.linnebacher@med.uni-rostock.de

**Keywords:** galvanotaxis, glioblastoma, brain metastasis, migration, capivasertib, afatinib

## Abstract

Galvanotaxis, the migration along direct current electrical fields, may contribute to the invasion of brain cancer cells in the tumor-surrounding tissue. We hypothesized that pharmacological perturbation of the epidermal growth factor (EGF) receptor and downstream phosphatidylinositol 3-kinase (PI3K)/AKT pathway prevent galvanotactic migration. In our study, patient-derived glioblastoma and brain metastases cells were exposed to direct current electrical field conditions. Velocity and direction of migration were estimated. To determine the effects of EGF receptor antagonist afatinib and AKT inhibitor capivasertib, assays of cell proliferation, apoptosis and immunoblot analyses were performed. Both inhibitors attenuated cell proliferation in a dose-dependent manner and induced apoptosis. We found that most of the glioblastoma cells migrated preferentially in an anodal direction, while brain metastases cells were unaffected by direct current stimulations. Afatinib presented only a mild attenuation of galvanotaxis. In contrast, capivasertib abolished the migration of glioblastoma cells without genetic alterations in the PI3K/AKT pathway, but not in cells harboring *PTEN* mutation. In these cells, an increase in the activation of ERK1/2 may in part substitute the inhibition of the AKT pathway. Overall, our data demonstrate that glioblastoma cells migrate in the electrical field and the PI3K/AKT pathway was found to be highly involved in galvanotaxis.

## 1. Introduction

High-grade gliomas have one of the worst survival prognoses of all common human tumor diseases. Even with multimodal approaches of surgical resection and a combined radiochemotherapy regime, average survival times of only 15–18 months after diagnosis are achieved [1,2,3]. One major reason for the poor prognosis and limited survival of current therapeutic approaches is the diffuse infiltration of glioblastoma cells in the tumor-surrounding tissue [4]. Cells outside the tumor bulk often escape surgical resection and are potentially outside of the target volume in radiotherapy regimen. The infiltrative migration of glioma cells is a complex process with multiple mechanisms involved [5]. To infiltrate the tumor-surrounding tissue, glioblastoma cells take advantage of the perivascular space of pre-existing blood vessels and white matter tracts [6].

One of the driving forces of invasion could be galvanotaxis (also referred to as electrotaxis)—the migration of cells along direct current (DC) electrical fields [7]. These low-voltage DC electrical fields emerge from asymmetric transmembrane ionic currents to generate the cellular membrane potential which in total forms transepithelial potentials [8,9]. As a result, the membrane of cells within the field will be hyperpolarized on the anodal side and depolarized on the cathodal one. Furthermore, our current understanding of galvanotaxis also assumes that membrane-bound components may be electrophoretically distributed via a process termed electromigration [10,11]. In the end, electromigration was associated with the clustering of membrane-bound receptors predominantly to one or the other side of the cells within the membrane. Both perturbation of the resting membrane potential and clustering of membrane-standing receptors may orchestrate the flux of ions—especially the influx of Ca^2+^ from the extracellular space—that triggers the process of migration [5].

Under physiological conditions, galvanotaxis was found to be involved in neural development and wound healing [12,13]. In the field of oncology, galvanotaxis was reported to be present in various tumor entities like breast cancer [14,15], lung cancer [16] and prostate carcinoma [17]. With respect to brain tumors, previous in vitro studies on glioma cell lines indicated that DC fields may also affect the directional migration of these tumor cells [18,19,20,21,22]. Furthermore, electrical stimulation activates signaling pathways shared with chemotactic stimuli like the phosphatidylinositol 3-kinase (PI3K)/AKT pathway downstream of receptor tyrosine kinases (RTK), e.g., epidermal growth factor (EGF) receptor or platelet-derived growth factor receptor A [19,20]. In glioblastoma, the EGF receptor is often found to be highly amplified and its expression is associated with tumorigenesis and tumor progression [23]. In high-grade glioma, genes encoding for RTK and members of the PI3K/AKT pathway are altered in up to 90% of cases [24,25]. This also holds true for one of the main negative regulators of this signaling pathway, phosphatase and tensin homolog (PTEN). Here, in ~40% of all glioblastoma samples, loss-of-function mutations were found [26,27].

Recently, Clancy et al. 2021 showed that galvanotactic migration was disturbed by upregulation of PTEN via PPARγ activation, underlining the importance of the PI3K/AKT pathway in the galvanotaxis of glioblastoma cells [22]. Remarkably, even in cells with PTEN mutations localized within the phosphatase domain, effects on the directedness of migration after PPARγ stimulation were described. Due to the limited number of studies with respect to glioma and glioblastoma, it remains unclear if the PI3K/AKT pathway downstream of the EGF receptor is a universal key player that orchestrates migration in electrical fields.

In our study, we systematically examined the galvanotactic migration of patient-derived low-passage cells derived from glioblastoma and brain metastases derived from surgically resected tissue. Furthermore, we focus on the EGF receptor and PI3K/AKT as potential key players in galvanotaxis.

## 2. Materials and Methods

### 2.1. Patient-Derived Low-Passage Cell Lines

In this study, five glioblastoma (WHO grade IV) and two brain metastasis low-passage cell lines were used. All procedures were approved by the Ethics Committee of the University Medicine of Rostock (reference ID: A 2009/34) in accordance with generally accepted guidelines for the use of human material. Tumors were obtained from surgery, with informed written patient consent. The establishment of the glioblastoma cell lines (referred to as HROG cell lines) from primary brain tumor specimens was described in detail in Mullins et al. 2013 [28]. Both metastasis cell lines (referred to as HROBM cell lines) were generated in the same way. HROG02, HROG15, and HROG24 were obtained from untreated primary tumors, while the HROG05 and HROG17 cell lines were established from relapsed glioblastoma (Table 1). All glioblastoma cell lines, except HROG24 presented methylated O6-methylguanine-DNA methyltransferase (MGMT) promoter regions. Instead, in HROG24 cells both copies of the MGMT gene are lost [29]. None of the glioblastoma cell lines harbor isocitrate dehydrogenase 1 (IDH1) mutations. With respect to molecular subclassification, HROG02 and HROG24 were identified as the proneural subtype, HROG05, HROG15 and HROG17 were classified as the mesenchymal subtype [29].

Brain metastasis cell lines HROBMC01 and HROBML01 were obtained from treatment-naive metastases of a 60-year-old female patient suffering from colorectal carcinoma and a 67-year-old male patient suffering from lung carcinoma, respectively.

All cell lines were cultured in Dulbecco’s Modified Eagle Medium: Nutrient Mixture F-12 (DMEM/F12; from PAN Biotech, Aidenbach, Germany) with 10% fetal calf serum (FCS, Bio&SELL, Feucht, Germany). Culturing of the cells was done at 37 °C in a 5% CO_2_ humidified atmosphere. In constant intervals, cell culture supernatants were tested for mycoplasma contamination employing MycoAlert Mycoplasma Detection Kit (Lonza, Basel, Switzerland). All experiments were performed with ≤50 cell passages.

### 2.2. Quantification of DNA Synthesis

To gauge the effects of the EGF receptor antagonist afatinib and pan-AKT inhibitor capivasertib (both from Selleck Chemicals, Houston, TX, USA) on DNA synthesis, a 5-bromo-2′-deoxyuridine (BrdU) incorporation assay kit (Roche Diagnostics GmbH, Mannheim, Germany) was employed. For this purpose, the cells (HROG cells: 1 × 10^3^/well; metastases cells: 3 × 10^3^/well) were seeded in 96-well half area microplates at equal densities and allowed to adhere overnight in complete culture medium. On the following day, the culture medium was substituted with a medium supplemented with small molecule inhibitors (afatinib or capivasertib) at the indicated doses. After an incubation period of 32 h, BrdU labeling was initiated by adding labeling solution at a final concentration of 10 µM. Another 16 h later, labeling was stopped, and BrdU uptake was measured according to the manufacturer’s instructions using a GloMax-Multi Detection System (Promega, Madison, WI, USA).

### 2.3. Caspase 3/7 Activation Assay

Caspase-3/7 activity was measured in a cell-based assay using the luminogenic substrate Z-DEVD-aminoluciferin with a tetrapeptide sequence specific for caspase-3/7. For this purpose, the glioblastoma (3 × 10^3^ cells/well) and brain metastasis cells (1 × 10^4^ cells/well) were seeded in 96-well plates in complete culture medium. The next day, either an inhibitor or the solvent was added at the indicated doses and incubation continued for an additional 6 h. Afterwards, caspase activity was measured following the instructions of the manufacturer (Promega). Briefly, the cell culture plates were removed from the incubator and luminogenic substrate was added. Assay plates were incubated at 22 °C for 1 h before recording the luminescence with a GloMax-Multi Detection System.

### 2.4. Migration in the Direct Current Electrical Field

To test the migratory behavior of brain tumor cells in electrical fields, custom-made DC chambers (Supplementary Figure S1A) were employed as described elsewhere [30]. Briefly, we took advantage of a DC chamber design that is based on a blueprint published by Yang and co-workers [31]. Each chamber was made of polyether ether ketone (PEEK) and consists of two parts. Prior to cell culture experiments, all chamber parts were cleaned with 70% ethanol, washed with a mild detergent, and rinsed excessively with distilled water before sterilization. After positioning a 24 × 60 mm coverslip in the upper part, chambers were screwed together and sterilized by UV light. Afterwards, the coverslips were coated with collagen I (Advanced Biomatrix, San Diego, CA, USA) for 1 h and were washed twice with PBS and cell culture medium. Next, the tumor cells (6 × 10^3^ glioblastoma or 8 × 10^3^ metastases cells per DC chamber) were seeded onto the coverslips and the DC chambers were sealed with a cover glass. After 24 h, the cells were washed twice and then sealed with fresh cell culture medium. Then, silver/silver chloride electrodes were placed into outer reservoirs with Ringer’s solution (Supplementary Figure S1A). Current flow was conducted by agar bridges consisting of 2% agarose (TopVision agarose, ThermoScientific, Waltham, MA, USA) in Ringer’s solution (Braun, Melsungen, Germany). A DC power supply (Standard Power PackP25, Biometra, Göttingen, Germany) was used to apply current for 6 h. Voltage was measured directly at the borders of the cell area with a 25 mm distance in between with a multimeter (Voltcraft VC220, Conrad Electronic, Wollerau, Switzerland) and adjusted during the experiments to maintain a constant electrical field strength. 

To estimate the position of the cells prior to and after 6 h of DC stimulation, micrographs of cells at eight fixed positions distributed over the whole area were taken at the beginning and end of the experiment employing a Leica DMI 6000 microscope (Leica, Wetzlar, Germany) with Leica Application Suite (v. 2.0.0.13332) software package. The exact overlay of both micrographs taken at the start and end was brought about using the image software GIMP (2.10.30) and these pictures were exported for evaluation of cell migration in ImageJ (1.53e). Four to five cells per field of view were analyzed by encircling cells including all cell extensions and centering cells (Supplementary Figure S1B). The circle center coordinates were determined at the start (*X*_0_/*Y*_0_) and 6 h later (*X*_1_/*Y*_1_). The distances of each dimension in the two-dimensional system and overall migration distance ($d=\sqrt{{\left(X_{0}-X_{1}\right)}^{2}+{\left(Y_{0}-Y_{1}\right)}^{2}}$) were calculated (referred to as the absolute value of migration). In most cases, X/Y position data of up to 40 cells per DC chamber could be obtained to calculate a mean migration of this DC chamber (i.e., one biological replicate). All *n*-numbers given in the text and figures correspond to the number of biological replicates.

To examine the effects of afatinib, capivasertib, or pan-AKT inhibitor MK-2206 (Selleck Chemicals) on HROG02, HROG15, and HROG17, cells in the DC electric field were treated for the entire duration of the experiment.

Electrical field strength may affect the DC-dependent migration of glioma cells [19,22]. In previous studies electric fields ranged from 10–300 V/m [18,21,22,32,33,34,35]. Therefore, in our experiments, well-established field strengths were used. In initial experiments, electrical field strengths ranging from 180 to 220 V/m (mean ~200 V/m) were applied. In these pilot experiments, no significant correlation of electrical field strength and migration velocity (*n* = 82 experiments; Pearson correlation coefficient was 0.18; *p* = 0.0907) or distance of migration along electrical force lines (e.g., in x-dimension; *n* = 82 experiments; Pearson correlation coefficient was 0.15; *p* = 0.166) was found. This allowed us to pool all biological replicates of one experimental group.

### 2.5. Immunoblotting

Glioblastoma cells (3 × 10^4^ cells/well) were seeded in 12-well plates in complete culture medium. After two days, the cells were treated with afatinib or capivasertib for 6 h. Afterwards, protein extracts were prepared and subjected to immunoblot analysis as previously described [36]. To receive total cellular protein, boiling lysis buffer (2% sodium dodecyl sulphate (SDS), 10% glycerol, 5 mM ethylenediaminetetraacetic acid (pH 8.0), 62.5 mM Tris-HCl (pH 6.8), 0.01% 3,3′,5,5′-tetrabromophenolsulfonphthalein, 5% 2-mercaptoethanol) was added directly to the cell monolayer. Protein crude extracts received from equal numbers of cells were separated by 10% SDS-polyacrylamide gel electrophoresis and blotted onto polyvinylidene fluoride membrane. Afterwards, membranes were blocked for 1 h using blocking buffer (10 mM Na_2_HPO_4_, 137 mM NaCl, 2 mM KH_2_PO_4_, 2.68 mM KCl, 0.05% Tween^®^ 20 (pH 7.4), 2% bovine serum albumin (Sigma, Taufkirchen, Germany), before primary antibodies were added, and incubation continued overnight. The following primary antibodies (all from Cell Signaling Technology, Frankfurt am Main, Germany, unless specified otherwise) were employed: anti-phospho (P)-AKT (P-AKT; #4060), anti-AKT protein (#4691), anti-phospho-ERK1/2 (P-ERK1/2) (#4370), anti-GAPDH (#2118), and anti-ERK1/2 (Abcam, Cambridge, UK; ab184699). The blots were developed using LI-COR reagents for an Odyssey Infrared Imaging System as described elsewhere [36]. The signal intensities of the investigated proteins were quantified by means of the Odyssey^®^ software (for single-lane fluorescence signal intensities see Supplementary Figure S6). Signals obtained for P-AKT, AKT protein, P-ERK1/2 and total ERK1/2 protein were normalized for loading differences by calculating the ratio to GAPDH. In a second step, P-AKT and P-ERK1/2 were normalized to total AKT and total ERK1/2 protein, respectively.

### 2.6. Statistical Analysis

Statistical analysis was performed with SigmaPlot 13.0. Experimental results are illustrated in box plots or given as mean ± standard error of the mean (SEM) for the indicated number of experiments. Mean group differences were tested for significance using the nonparametric Kruskal–Wallis test before for multiple comparisons subgroups were tested with post hoc Dunn’s test. Comparisons of two independent groups were performed with Mann–Whitney U test. For the analysis of migration velocity and direction, a two-way ANOVA followed by Bonferroni *t*-test was used. A significance level of *p* < 0.05 was considered to be statistically significant.

## 3. Results

### 3.1. Glioblastoma Cells Migrate in the Electrical Direct Current Field

**Figure 1:**
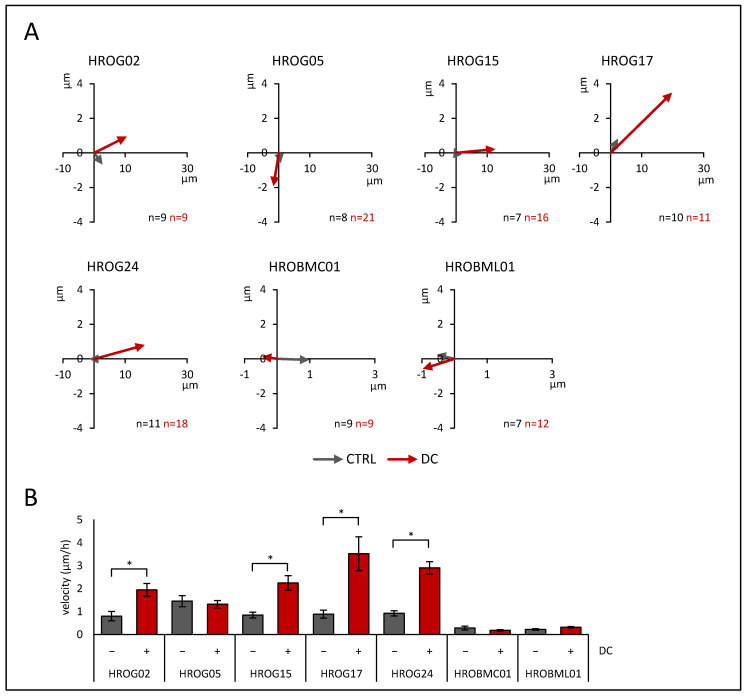
Migration in the DC electrical field of glioblastoma cells (HROG02, HROG05, HROG15, HROG17, and HROG24) and brain metastases cells (HROBMC01 and HROBML01). Tumor cells were seeded on collagen-coated coverslips that were mounted in DC chambers. Position of cells after DC stimulation was estimated as described in the Materials and Methods section in detail. (**A**) Sum vectors of migration distance after 6 h of DC stimulation (red) and control cultures (CTRL, gray). Please note different scaling of x-axis between glioblastoma and brain metastases cells. Positive values in x-dimension imply anodal migration. (**B**) Absolute value of the mean velocity of migration ± DC (mean: 200 V/m). Data are presented as mean ± SEM (*n* = 7–21 separate biological replicates; up to 40 cells were analyzed per biological replicate); * *p* < 0.05 versus control cultures w/o DC (Mann–Whitney U test).

In initial experiments, five patient-derived low-passage glioblastoma cell lines and two cell lines derived from brain metastases were tested for their migratory behavior in the direct current (DC) electrical field. As illustrated by the gray sum vectors in Figure 1A, all cell lines presented a migratory phenotype even in the absence of an electrical field (referred to as control (CTRL); see Supplementary Figures S2–S5 for single cell data). However, when the cells were exposed to DC electrical fields, glioblastoma cell lines HROG02 (CTRL: 0.79 ± 0.21 µm/h vs. DC: 1.94 ± 0.28 µm/h), HROG15 (CTRL: 0.84 ± 0.13 µm/h vs. DC: 2.25 ± 0.32 µm/h), HROG17 (CTRL: 0.88 ± 0.18 µm/h vs. DC: 3.5 ± 0.74 µm/h), and HROG24 (CTRL: 0.92 ± 0.11 µm/h vs. DC: 2.91 ± 0.27 µm/h) showed significantly enhanced migration velocities (*p* < 0.05 for all, U test; Figure 1B). In contrast, HROG05 (CTRL: 1.45 ± 0.24 µm/h vs. DC: 1.32 ± 0.15 µm/h) and both metastases cell lines HROBMC01 (CTRL: 0.28 ± 0.08 µm/h vs. DC: 0.18 ± 0.03 µm/h) and HROBML01 (CTRL: 0.22 ± 0.03 µm/h vs. DC: 0.31 ± 0.03 µm/h) were unaffected by the DC electrical field.

Additionally, a two-way ANOVA (factor tumor origin, i.e., glioblastoma vs. metastases and factor current, i.e., CTRL vs. DC) with Bonferroni post hoc test revealed that glioblastoma cells exhibited a significantly higher migration velocity than metastasis cell cultures (*p* < 0.001). Furthermore, stimulation by DC significantly enhanced the velocity of all cultures (*p* = 0.002), but not in the subgroup of metastases (*p* = 0.991; two-way ANOVA followed by Bonferroni *t*-test).

**Figure 2:**
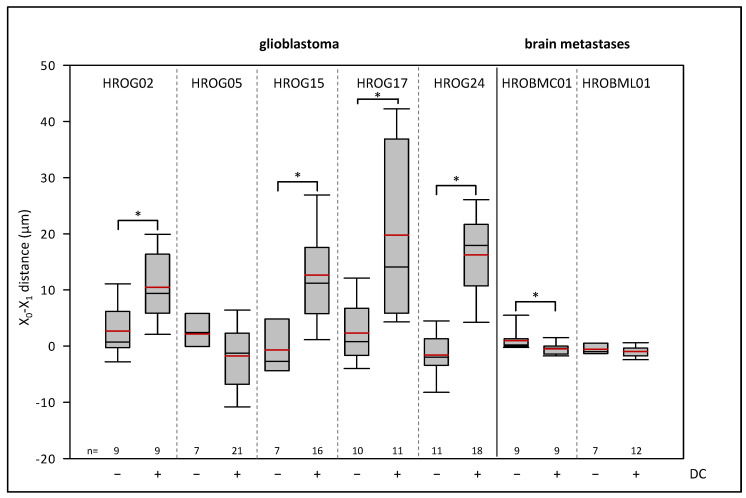
Effects of DC electrical fields on pole-directed migration of glioblastoma and brain metastases cells. Glioblastoma and brain metastases cells were seeded on collagen-coated coverslips, and these were mounted in DC chambers. Pole-directed migration was calculated after 6 h of DC stimulation. Median is shown as a black colored line and the mean in red. * *p* < 0.05 versus control cultures w/o DC (Mann–Whitney U test). For comparison of glioblastoma cells and metastases cells, see text.

Next, we examined whether the cells migrated preferentially in the anodal or cathodal direction under DC conditions. Therefore, the distance of migration in the x-dimension from the beginning to the end of the experiment was calculated (Figure 2). Glioblastoma cell lines HROG02 (CTRL: 2.68 ± 1.45 µm vs. DC: 10.46 ± 2.03 µm), HROG15 (CTRL: −0.67 ± 1.73 µm vs. DC: 12.65 ± 2.09 µm), HROG17 (CTRL: 2.32 ± 1.66 µm vs. DC: 19.76 ± 4.55 µm), and HROG24 (CTRL: −1.57 ± 1.19 µm vs. DC: 16.24 ± 1.90 µm) significantly migrated in the anodal direction (e.g., to the positive pole), whereas in cultures of HROG05 (CTRL: 2.15 ± 1.77 µm vs. DC: −1.74 ± 1.44 µm) and HROBML01 (CTRL: −0.55 ± 0.40 µm vs. DC: −0.97 ± 0.28 µm) no changes were found (*p* < 0.05, U test). Notably, HROBMC01 cells presented a cathodal migration (CTRL: 0.97 ± 0.61 µm vs. DC: −0.47 ± 0.33 µm).

We further asked whether glioblastoma and metastases cells might differ in their pole-directed migration. For this purpose, a two-way ANOVA was performed (factor tumor, i.e., glioblastoma vs. metastases, and factor current, i.e., CTRL vs. DC). Overall, there was an increased migration of the cells towards the anode within the electric field (*p* = 0.013). A comparison of both tumor groups revealed a significantly larger anodal migration of glioblastoma cells than in metastases cells under DC conditions (*p* < 0.001), while there were no differences under control conditions (*p* = 0.809).

### 3.2. Capivasertib and Afatinib Affect Cell Proliferation and Cell Survival of Glioblastoma and Brain Metastases Cells

**Figure 3:**
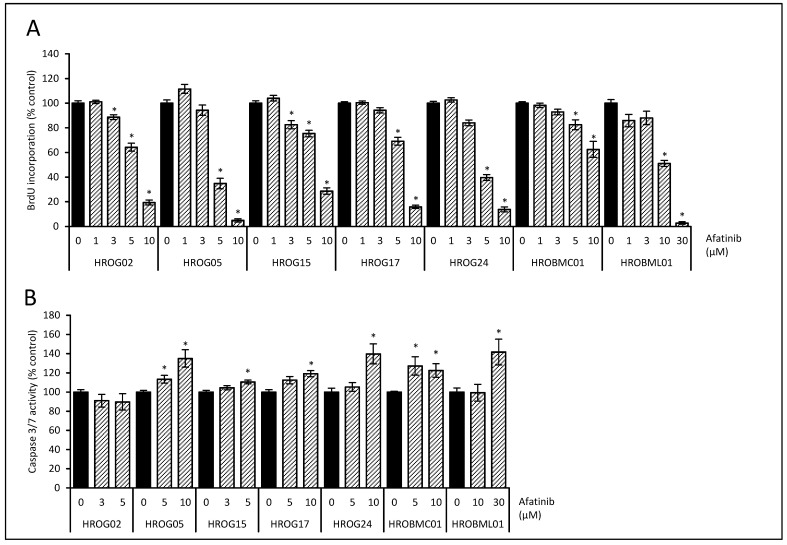
Effects of the EGF receptor inhibitor afatinib on cell proliferation and apoptosis. (**A**) Glioblastoma cells (HROG02, HROG05, HROG15, HROG17, and HROG24) and brain metastasis cell (HROBMC01 and HROBML01) growing in 96-well half-area microplates were treated with afatinib (striated bars) or solvent (black bars) for 48 h, before DNA synthesis was assessed with the BrdU incorporation assay. One hundred percent BrdU incorporation corresponds to cells cultured with solvent only. Data are presented as mean ± SEM (*n* ≥ 18 separate cultures); * *p* < 0.05 versus control cultures (Kruskal–Wallis test with post hoc Dunn’s test). (**B**) Subconfluent-growing glioblastoma cells were challenged with afatinib (striated bars) or solvent control (black bars) for 6 h followed by caspase 3/7 enzyme activity quantification. Data are presented as mean ± SEM (*n* ≥ 9 separate cultures for caspase activity assay); * *p* < 0.05 versus control cultures (Kruskal–Wallis test with post hoc Dunn’s test).

Since we aimed to contribute to a better understanding of molecular mechanisms of galvanotaxis in brain tumors, we investigated the effects of inhibitors that interfere with possible key molecules. Whereas previous studies highlight the impact of the PI3K/AKT pathway on galvanotactic behavior [13,37], the role of the EGF receptor is a matter of debate. To address this issue, the biological effects of the EGF receptor antagonist afatinib and the ATP-competitive AKT inhibitor capivasertib on proliferation were determined. In addition, cell death via apoptosis was assessed by caspase 3/7 activation. In all cell lines, afatinib inhibited proliferation in a dose-dependent manner (Figure 3A). Next, caspase 3/7 activity was investigated with doses of afatinib that achieved a significant reduction of proliferation in BrdU assays. In all but one cell line (HROG02), a moderate, but significant increase in caspase 3/7 enzyme activity after afatinib treatment was observed (Figure 3B).

**Figure 4:**
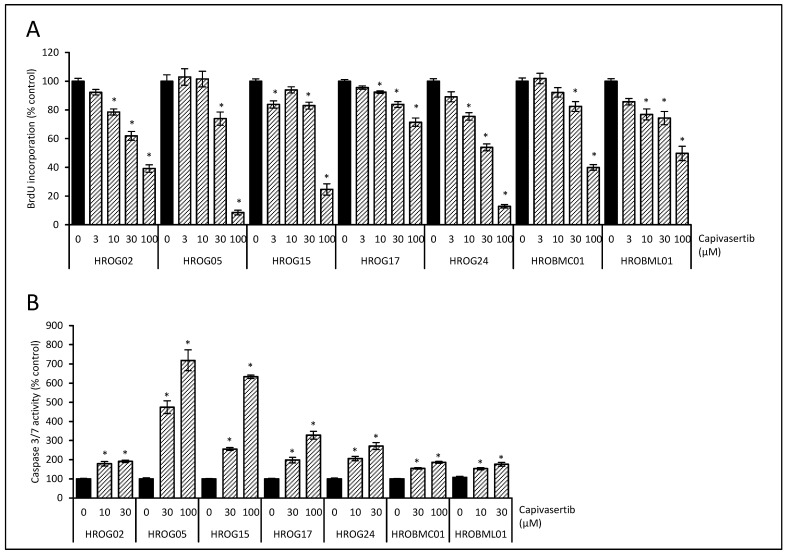
Effects of the AKT inhibitor capivasertib on cell proliferation and apoptosis. (**A**) Low-passage glioblastoma cells (HROG02, HROG05, HROG15, HROG17, and HROG24) and brain metastasis cells (HROBMC01 and HROBML01) growing in 96-well half-area microplates were treated with capivasertib (striated bars) or solvent (black bars) for 48 h, before DNA synthesis was assessed with the BrdU incorporation assay. One hundred percent BrdU incorporation corresponds to cells cultured with solvent only. Data are presented as mean ± SEM (*n* ≥ 18 separate cultures); * *p* < 0.05 versus control cultures (Kruskal–Wallis test with post hoc Dunn’s test). (**B**) Subconfluent-growing glioblastoma cells were challenged with capivasertib (striated bars) or solvent (black bars) for 6 h followed by caspase 3/7 enzyme activity quantification. Data are presented as mean ± SEM (*n* ≥ 9 separate cultures for caspase activity assay); * *p* < 0.05 versus control cultures (Kruskal–Wallis test with post hoc Dunn’s test).

In parallel approaches, exposure of the cells with capivasertib led to a dose-dependent reduction of BrdU incorporation in all cell lines (Figure 4A). In subsequent experiments, all selected doses of the AKT inhibitor led to a significant increase in the activation of caspase 3/7 (Figure 4B). Based on these data, doses of afatinib and capivasertib were selected for subsequent examination of the migration in DC electrical field experiments.

### 3.3. Capivasertib Inhibites Galvanotaxis of PTEN Wild-Type Glioblastoma Cells

Next, we asked whether capivasertib or afatinib might impair the galvanotactic migration of brain tumor cells. To this end, we focused on three glioblastoma cell lines that in our experiments so far presented a phenotype with anodal migration (Figure 2). On the molecular level, the low-passage cell lines differed in their genetic status of *PTEN*, one of the major negative regulators of the PI3K/AKT signaling pathway [38]. While HROG02 presented no alterations in *PTEN*, HROG15 (PTEN S170N) and HROG17 (PTEN R130*) harbor alterations in hotspot regions of the gene [29]. Both mutations led to an impaired phosphatase activity of the enzyme [39].

**Figure 5:**
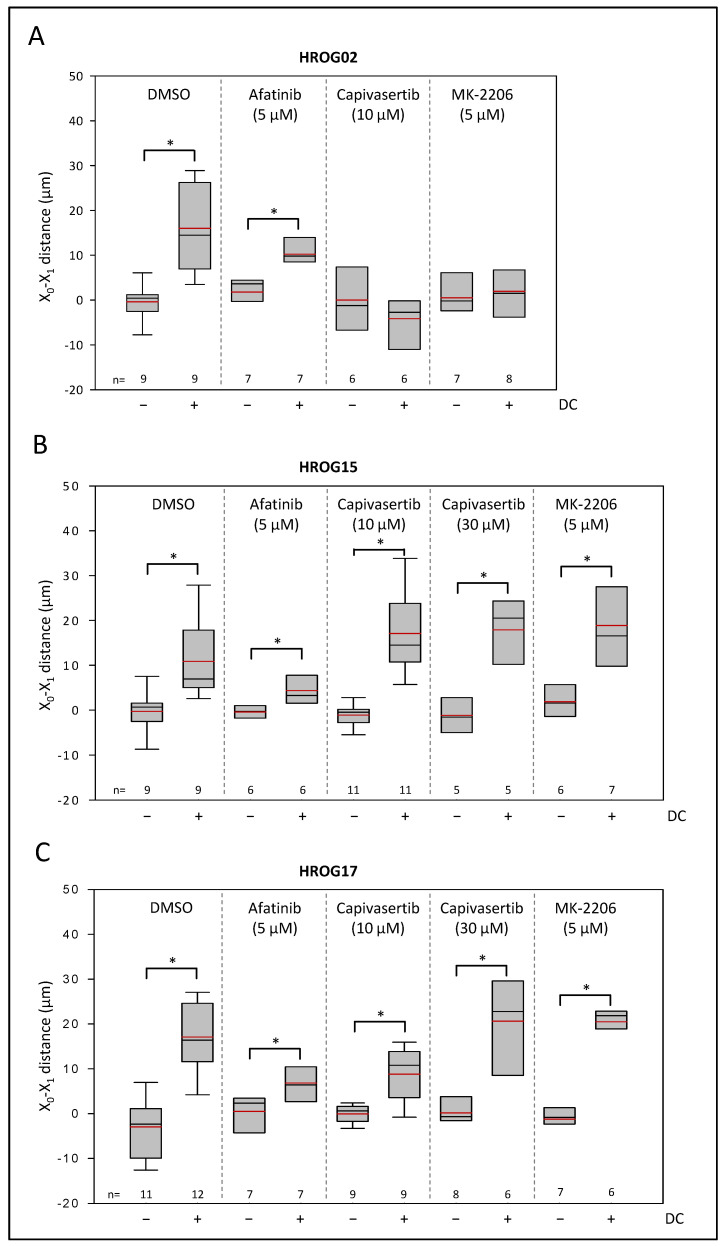
Effects of AKT inhibitors and afatinib on galvanotactic migration of (**A**) HROG02, (**B**) HROG15, and (**C**) HROG17. Glioblastoma cells growing on coverslips were treated with afatinib, capivasertib, MK-2206, or solvent (DMSO) at the indicated doses. Median is shown as a black colored line and the mean as a red line. Data are based on 5–12 independent biological replicates (in each replicate migration of up to 40 cells was analyzed), * *p* < 0.05 versus control cultures w/o DC (Mann–Whitney U test).

As illustrated in Figure 5, none of the three cell lines were affected in the migratory behavior by EGF receptor antagonist afatinib (*p* < 0.05, U test). Afatinib could not prevent anodal migration, but a two-way ANOVA (factor cell line, i.e., HROG02, HROG15, and HROG17, and factor treatment, i.e., DMSO vs. afatinib) revealed that the EGF receptor antagonist may attenuate the distance of the anodal migration. Here, cultures treated with afatinib under DC conditions presented an anodal migration distance reduced to 36 ± 6% in comparison to DC controls (*p* < 0.05, two-way ANOVA followed by Bonferroni *t*-test). No significant differences between HROG cell lines were found.

In marked contrast, anodal movement of HROG02 was abolished by exposure of the cells to 10 µM capivasertib (Figure 5). Notably, the effect of capivasertib was also achieved by exposure of the HROG02 cells to allosteric AKT inhibitor MK-2206 (5 µM; dose based on [40] and own BrdU studies (data not shown); Figure 5A). However, HROG15 and HROG17 cells were unaffected by 10 µM capivasertib and even a high dose of 30 µM had no effect on the anodal migration of the cells (Figure 5B,C). Again, treatment of the cells with MK-2206 matched the effects of capivasertib in both cell lines.

**Figure 6:**
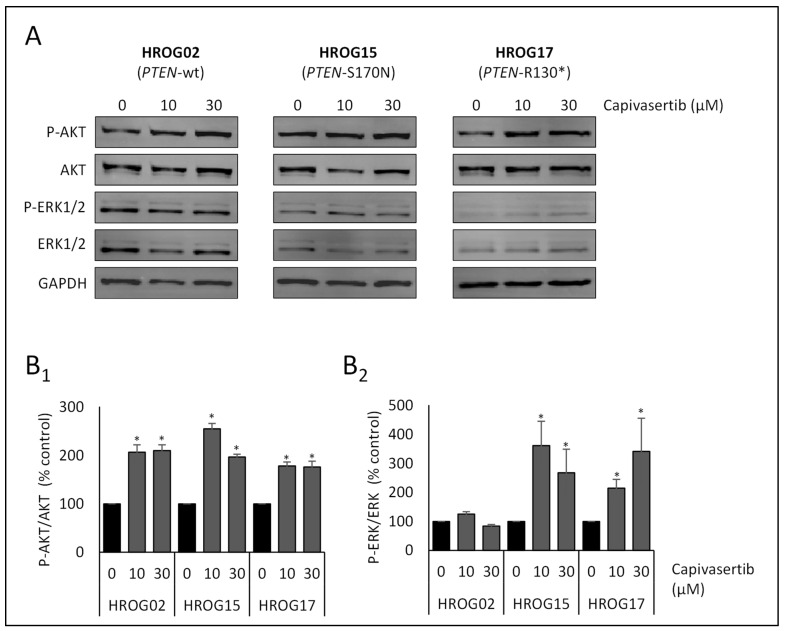
Effects of capivasertib on the phosphorylation of AKT and ERK (i.e., ERK1/2) in glioblastoma cell lines. The cells were grown in 12-well plates before the culture medium was supplemented with capivasertib at the indicated concentrations. Control cultures were treated with solvent (DMSO) only. After an incubation period of 6 h, protein extracts from equal amounts of cells were subjected to immunoblot analysis. P-AKT and P-ERK, the respective total proteins, and GAPDH (for loading control) were detected using fluorescein-labelled secondary antibodies. (**A**) For each cell line, one representative blot is shown. Subsequently, the ratios (**B_1_**) P-AKT/AKT protein ratios and (**B_2_**) P-ERK/ERK protein ratios were determined. Data from 6 independent experiments were used to calculate mean values ± SEM, * *p* < 0.05 versus control cultures (U test). Note that in cell lines without *PTEN* mutation no change in the phosphorylation of ERK was determined. In contrast, cells harboring *PTEN* mutations presented increased levels of phosphorylated proteins.

To obtain more detailed information on the molecular effects of capivasertib on the PI3K/AKT pathway, the status of phosphorylation of AKT as a surrogate marker for enzyme activation was estimated. Additionally, ERK1/2 as the effector kinase of the closely linked Raf-MEK-ERK pathway that contributes to cell migration [41] was also included in the immunoblot analyses. In all three cell lines, capivasertib increased the ratio of phosphorylated AKT (Ser473) to total AKT protein (*p* < 0.05, U test; Figure 6), which reflects the mechanism of action of the drug [42,43] and counts as a readout for an effective impairment of the kinase. With respect to the ERK1/2 signaling pathway, HROG02 showed no change in the phosphorylation of the protein (Figure 6). Interestingly, in HROG15 and HROG17 an increase in the phospho-ERK1/2 level could be observed after capivasertib treatment (*p* < 0.05, U test).

## 4. Discussion

The unmatched infiltrative migration of high-grade glioma cells remains a major challenge in the attenuation of the tumor progression and prolongation of patient survival. While most research in the past focused on mechanisms of migration in the microenvironment based on chemotaxis with aberrant glutamate signaling and disturbed intracellular Ca^2+^ homeostasis as one of the main factors [5,44], the migration along DC electrical fields within the brain has been almost neglected so far. In this study, we asked the questions of whether low-passage patient-derived brain cancer cells present a uniform migratory phenotype in the DC electrical field and how galvanotactic migration of tumor cells could be interrupted.

We found that patient-derived, low-passage glioblastoma cells were stimulated to a directional migration by DC electrical fields, confirming recently reported data [22] in line with previous publications focusing on glioblastoma cells [19,20,21,37]. 

In the current study, in principle, the cells may migrate preferentially to the cathode, the anode or show no directness after DC stimulation. The question of why cells prefer to migrate to one pole or the other has not yet been conclusively clarified, as several biophysical mechanisms seem to contribute to the overall outcome [7,45,46]. A preferred cathodal migration is believed to be based on anodal hyperpolarisation of the plasma membrane and depolarisation on the opposite side due to an asymmetric ion influx via voltage-gated ion channels [21] and ion channels like Kir4.2 that affect the resting membrane potential [18]. In addition, hyperpolarization of the plasma membrane attracts intracellular Ca^2+^ by passive electrochemical diffusion, which may increase the Ca^2+^ concentration on the anodal side of the cell, and thus may substantially contribute to the ion distribution in the cell [45]. Furthermore, the electrophoretic distribution of membrane-bound receptors and ion channels to different sides of the cells within the DC electrical field is a plausible hypothesis that may contribute to anodal or cathodal migration [16]. 

Interestingly, in contrast to the study of Clancy et al. 2021, in our hands, the cell line HROG05 was unaffected by DC stimulation [22]. One may speculate that culturing conditions and the number of cell passages may contribute to a differential response, as the authors demonstrated that depending on the culture conditions, HROG cells migrate in an anodal or cathodal direction.

Furthermore, we asked whether metastases cells whose primary tumor is outside the CNS showed a similar migration behavior as glioblastoma cells. Both lung cancer and colon cancer-derived cells presented an overall lower migratory phenotype in comparison to glioblastoma cells. Moreover, migration was not stimulated by DC electrical field conditions. Remarkably, studies based on established cell lines of lung cancer showed that these tumor models could be stimulated by DC electrical fields, with a cathodal migration being primarily observed [16,47,48,49], but anodal movement was also detected [50]. To the best of our knowledge, no data on galvanotaxis of colon cancer cells have been published so far. With respect to the enteric nervous system and the voltage-controlled contraction of smooth muscle in the gut, locally present low-voltage electric fields could facilitate galvanotaxis. In addition, colon carcinomas are characterized by altered expression of calcium-permeable channels [51], which may contribute to the migration in the electric field [21,52,53].

For the first time, we showed that the AKT inhibitor capivasertib inhibited proliferation and acted as an inducer of apoptosis in glioblastoma and brain metastases cells. A recently published study by Schmid et al. 2020 found a benefit with respect to overall survival and progression-free survival of patients with triple-negative breast cancer [54], a disease in which cancer cells respond to DC stimuli in vitro [14,15].

However, our major finding was that glioblastoma cells harboring an alteration in the phosphatase domain of *PTEN*, one of the main negative regulators of the PI3K/AKT pathway, were sensitive to AKT inhibition by capivasertib with respect to cell proliferation and cell survival, but not to galvanotactic migration. Additional experiments with the AKT inhibitor MK-2206 confirmed the effects of capivasertib on galvanotaxis. In contrast, galvanotactic migration of cells with the *PTEN* wild-type was abolished by AKT inhibition. Lyon et al. 2019 employed U-87 MG spheroids, a cell line with mutated *PTEN* [55], as a glioma model [20]. In contrast to our study, inhibition of AKT by MK-2206 abolished galvanotactic migration. However, in comparison to low-passage cell lines, long-term cultured U-87 MG cells present an overall lower response to DC electrical fields and may not be the most appropriate model to study galvanotaxis [19].

Intriguingly, in the two cell lines that were unaffected with respect to migration, an increase in the activation of ERK1/2 was observed, which may in part substitute the inhibition of the PI3K/AKT axis. In brain tumor-initiating cells of glioblastoma, Huang et al. 2016 showed that inhibition of PI3K results in a decreased migration towards the anode, but overall motility of the cells was not affected. Remarkably, the ERK inhibitor U0126 led to the opposite effect—decreased motility, but migration in the anodal direction was not disturbed [19]. As both PI3K/AKT and Raf-MEK-ERK signaling pathways are closely linked [56,57,58], an additional inhibition of ERK1/2 may result in an attenuation of migration in AKT-inhibitor-resistant cell lines.

In all three glioblastoma cell lines subjected to immunoblot analyses, capivasertib treatment led to an increase in the phosphorylation level of AKT. At a first glance, this seems to be counterintuitive as hyperphosphorylation of a kinase is known to be related to increased enzyme activity. However, capivasertib acts as an ATP-competitive inhibitor of all three isoforms of AKT [42,59]. While allosteric AKT inhibitors like MK-2206 prevent the phosphorylation of the kinase by keeping AKT in an inactive conformational state, ATP-competitive inhibitors lock AKT in the phosphorylated (e.g., Thr308 and Ser473) but non-functional state [60]. As access to phosphorylated amino acid residues is restricted, dephosphorylation of AKT by phosphatases like PP2A and PHLPP is attenuated [61].

Another finding was that the EGF receptor antagonist afatinib inhibited cell proliferation of glioblastoma cells [62,63,64]. Moreover, our data showed that metastasis cells were less susceptible, and therefore higher doses of the inhibitor were needed to effectively disturb cell growth. Unfortunately, while a case report from 2015 achieved a much longer patient survival with the add-on of afatinib in glioblastoma [65], further studies published less optimistic results [66,67]. Our data confirmed that inhibition of the EGF receptor had only a little effect on galvanotaxis as investigated in low-passage glioblastoma [19] and U-87 MG cells [20]. However, U-87 MG cells were in part susceptible to HER2–4 receptor inhibition [20]. The authors speculated that an asymmetrical distribution by electrophoresis contributed to those findings. In other tumor entities showing a galvanotactic phenotype, different results were found. In comparison to glioblastoma, in the cells of lung and breast cancer, a functional EGF receptor seemed to be an essential part of the response to the electrical field [16,68], while in CL 1–5 lung adenocarcinoma cells no effect by EGF receptor inhibition was observed [50].

To this end, we and others have investigated galvanotaxis of brain tumor cells in in vitro experiments only. As migration of tumor cells is believed to be a multifactorial process, it would not be unlikely if further aspects like the composition of the extracellular matrix and interaction with tumor surrounding cells highly contribute to a galvanotactic phenotype. Based on our data, we suggest follow-up studies employing animal models [34] adapted to brain cancers as a starting point to further evaluate the proposed mechanisms and to challenge key players that have been identified as essential in galvanotaxis.

## 5. Conclusions

Altogether, we can confirm that low-passage glioblastoma cells presented a galvanotactic phenotype, whereas cells from brain metastases were resistant to DC stimulation. The AKT inhibitor capivasertib attenuated cell proliferation and induced apoptosis in all tested glioblastoma cells, but failed to block migration in cells harboring a *PTEN* mutation. The EGF receptor antagonist afatinib mediated only weak attenuating effects on galvanotaxis of glioblastoma cells.

## Figures and Tables

**Table 1:** Tumor cell lines.

Tumor ID	Gender/Age	Tumor Location	Tumor Species (Origin)
HROG02	M/68	R; parietooccipital	primary glioblastoma
HROG05	F/60	L; temporal	relapsed primary glioblastoma
HROG15	M/56	R; parietal	primary glioblastoma
HROG17	M/70	L; parietooccipital	relapsed primary glioblastoma
HROG24	F/73	L; occipital	primary glioblastoma
HROBMC01	F/60	cerebrum	colon carcinoma
HROBML01	M/67	cerebrum	non-small cell lung cancer

## Data Availability

The data presented in this study are available on request from the corresponding author.

## Supplementary Materials

The following supporting information can be downloaded at: https://www.mdpi.com/article/10.3390/life12040580/s1, Figure S1. [A] DC stimulation chamber were used to investigate galvanotactic migration of glioblastoma and brain metastases cells. [B] Estimation of the two-dimensional migration (x,y) within the DC electrical field after 6 h of continuous stimulation. Figure S2. Migration of [A_1_] HROG02 and [B_1_] HROG05 glioblastoma cells after 6 h of DC electrical field stimulation is shown in red. Control cultures without DC are marked in black. Position of each cell is plotted in reference to the start of the experiment. [A2] and [B2] illustrate direction of galvanotactic migration ± DC. Figure S3. Migration of [A_1_] HROG15 and [B_1_] HROG17 glioblastoma cells after 6 h of DC electrical field stimulation is shown in red. Control cultures without DC are marked in black. Position of each cell is plotted in reference to the start of the experiment. [A_2_] and [B_2_] illustrate direction of galvanotactic migration ± DC. Figure S4. Migration of [A_1_] HROG24 glioblastoma cells after 6 h of DC electrical field stimulation is shown in red. Control cultures without DC are marked in black. Position of each cell is plotted in reference to the start of the experiment. [A_2_] illustrate direction of galvanotactic migration ± DC. Figure S5. Migration of [A_1_] HROBMC01 and [B_1_] HROBML01 metastases cells after 6 h of DC electrical field stimulation is shown in red. Control cultures without DC are marked in black. Position of each cell is plotted in reference to the start of the experiment. [A_2_] and [B_2_] illustrate direction of galvanotactic migration ±DC. Figure S6. Sample PVDF membranes of capivasertib effects on AKT and ERK1/2 activation. Precision Plus Protein Dual Color Standards (BIO-RAD) was used as molecular size marker. The blots were developed using LI-COR reagents for an Odyssey Infrared Imaging System and signal intensities of the investigated proteins were quantified by means of the Odyssey^®^ software (Table S1). For further details see the Materials and Methods section in the manuscript. Table S1. Signal intensities from each protein band were quantified in immunoblot analyses.
