# Supplementary material for: Galvanotactic Migration of Glioblastoma and Brain Metastases Cells
Source: Life (Basel). 2022 Apr 14;12(4):580. doi: 10.3390/life12040580 (PMC9027426; doi:10.3390/life12040580)
Supplement: Supplementary file 1 [file life-12-00580-s001.zip › life-1669967-supplementary.pdf]

life

Article

Galvanotactic Migration of Glioblastoma and Brain Metastases Cells

Falko Lange [1,2,*], Jakob Venus [1], Daria Shams Esfand Abady [1], Katrin Porath [1], Anne Einsle [1], Tina Sellmann [1], Valentin Neubert [1], Gesine Reichart [1], Michael Linnebacher [3], Rüdiger Köhling [1,2,†] and Timo Kirschstein [1,2,†]

1 Oscar-Langendorff-Institute of Physiology, Rostock University Medical Center, 18057 Rostock, Germany; jakob.venus@uni-rostock.de (J.V.); daria.abady@uni-rostock.de (D.S.E.A.); katrin.porath@uni-rostock.de (K.P.); anne.einsle@med.uni-rostock.de (A.E.); tina.sellmann@uni-rostock.de (T.S.); valentin.neubert@uni-rostock.de (V.N.); gesine.reichart@uni-rostock.de (G.R.); ruediger.koehling@uni-rostock.de (R.K.); timo.kirschstein@uni-rostock.de (T.K.)
2 Center for Transdisciplinary Neurosciences Rostock, University of Rostock, 18147 Rostock, Germany
3 Molecular Oncology and Immunotherapy, Rostock University Medical Center, 18057 Rostock, Germany; michael.linnebacher@med.uni-rostock.de
* Correspondence: falko.lange@uni-rostock.de
† These authors contributed equally to this work.

Supplementary

A

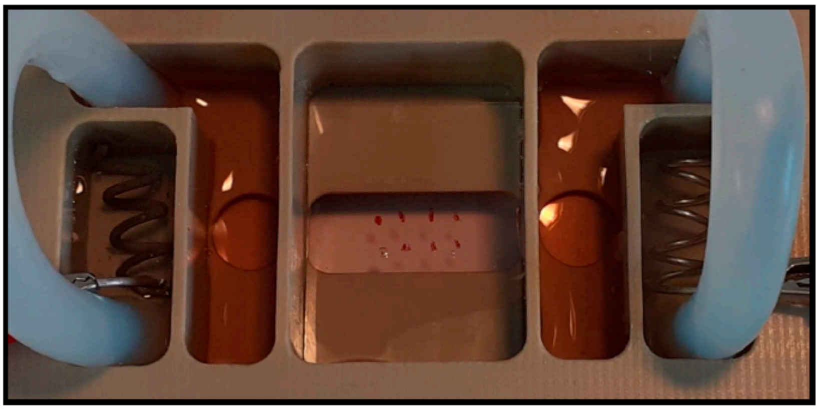

B

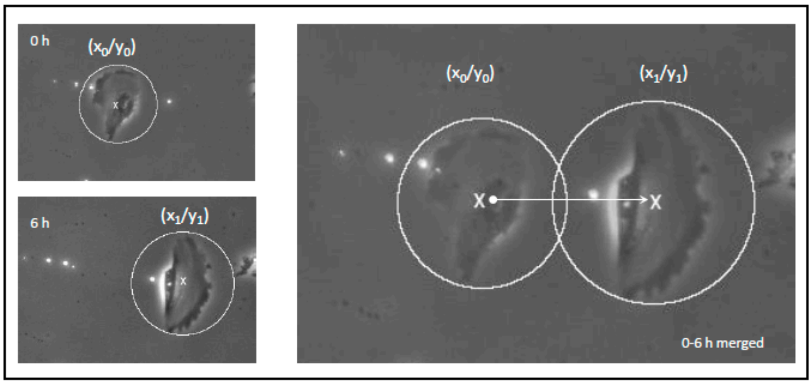

Supplementary Figure S1:[**A**] DC stimulation chamber were used to investigate galvanotactic migration of glioblastoma and brain metastases cells. [**B**] Estimation of the two-dimensional migration (x,y) within the DC electrical field after 6 hours of continuous stimulation.

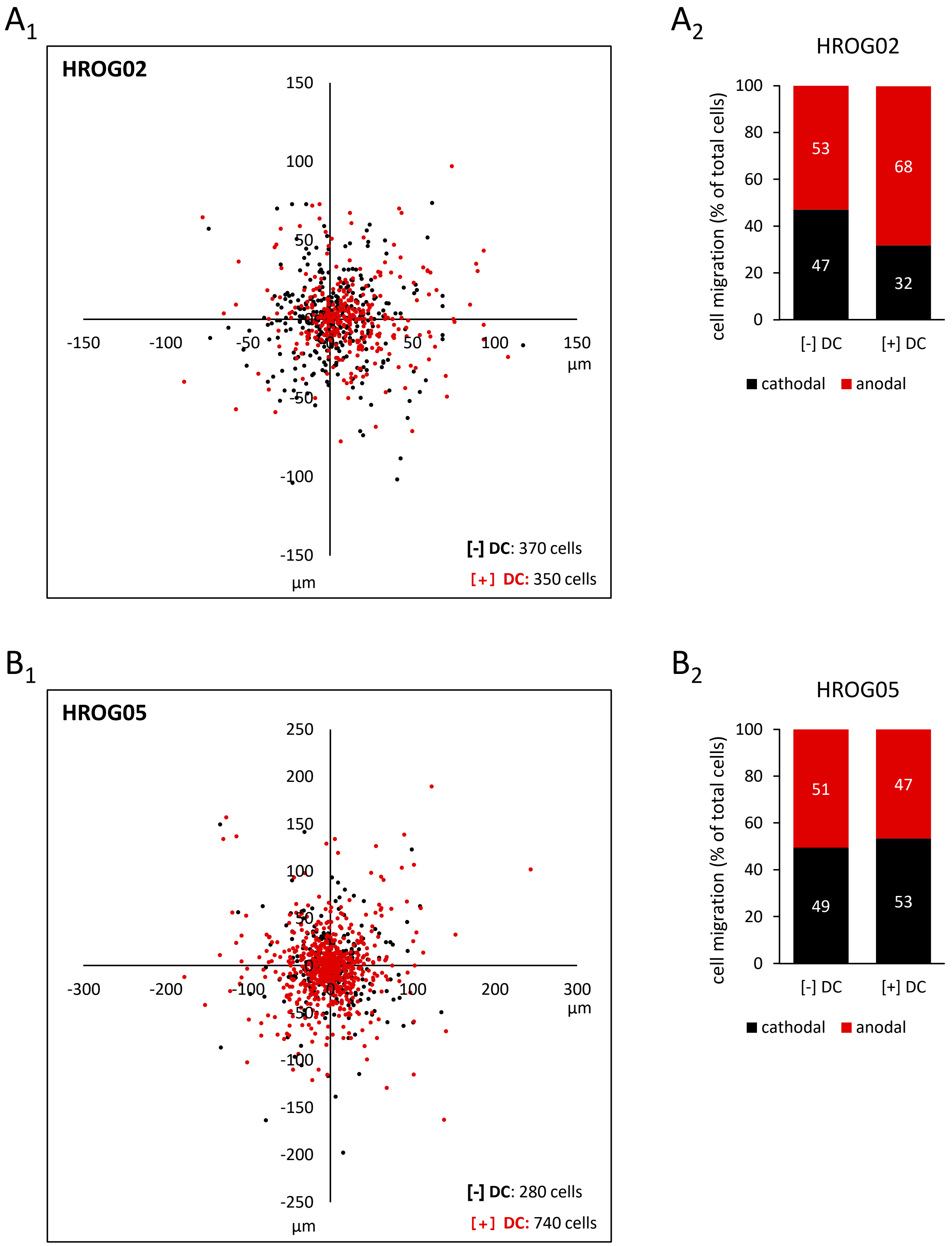

Supplementary Figure S2: Migration of [A_1] HROG02 and [B_1] HROG05 glioblastoma cells after 6 hours of DC electrical field stimulation is shown in red. Control cultures without DC are marked in black. Position of each cell is plotted in reference to the start of the experiment. [**A2**] and [**B2**] illustrate direction of galvanotactic migration ±DC.

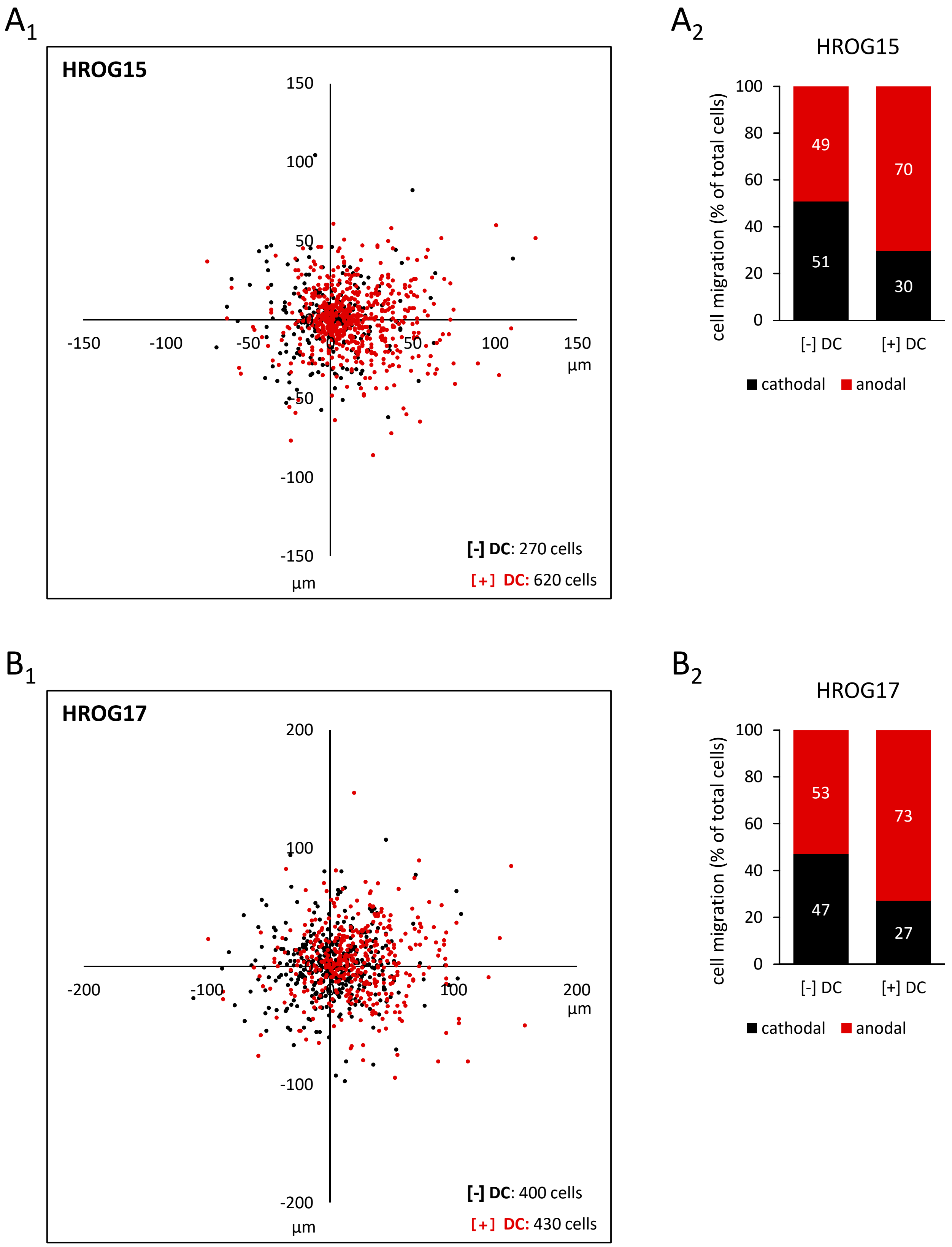

Supplementary Figure S3: Migration of [$\mathbf{A}_1$] HROG15 and [$\mathbf{B}_1$] HROG17 glioblastoma cells after 6 hours of DC electrical field stimulation is shown in red. Control cultures without DC are marked in black. Position of each cell is plotted in reference to the start of the experiment. [$\mathbf{A}_2$] and [$\mathbf{B}_2$] illustrate direction of galvanotactic migration ±DC.

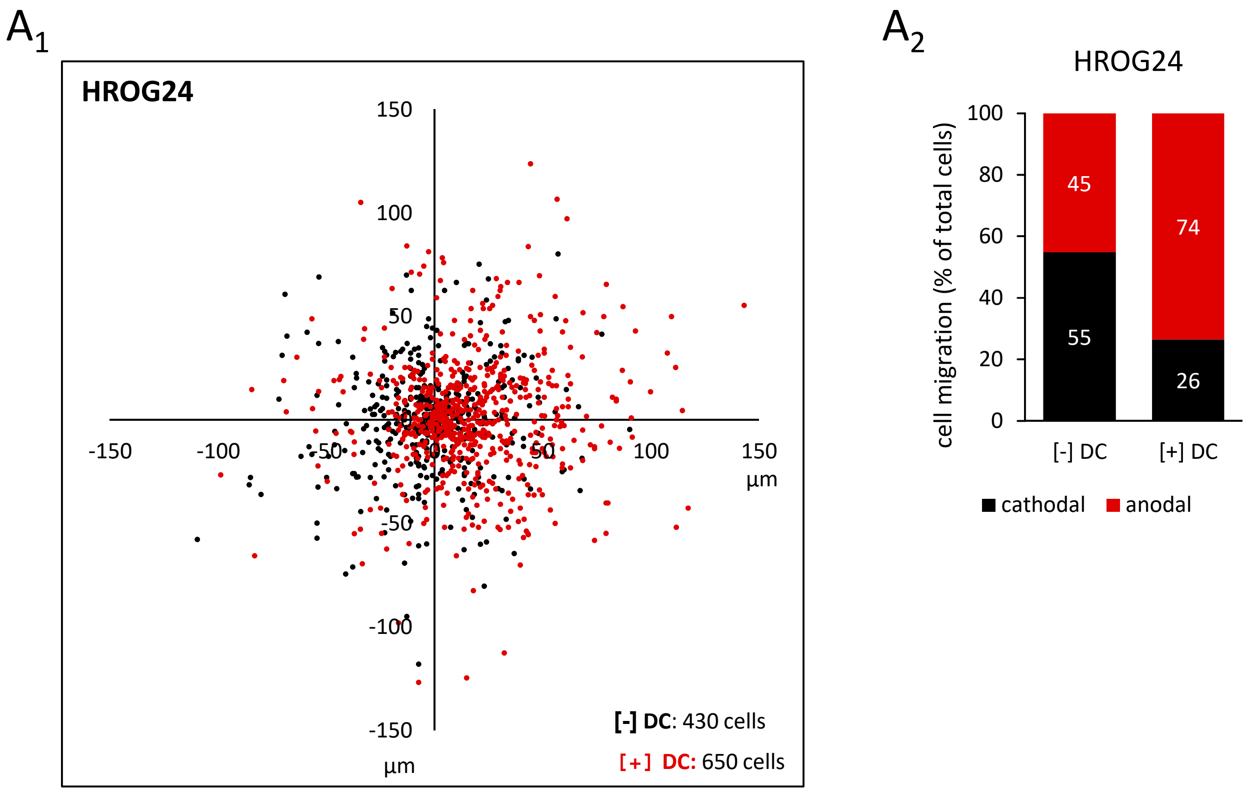

Supplementary Figure S4: Migration of [$\mathbf{A_1}$] HROG24 glioblastoma cells after 6 hours of DC electrical field stimulation is shown in red. Control cultures without DC are marked in black. Position of each cell is plotted in reference to the start of the experiment. [$\mathbf{A_2}$] illustrate direction of galvanotactic migration ±DC.

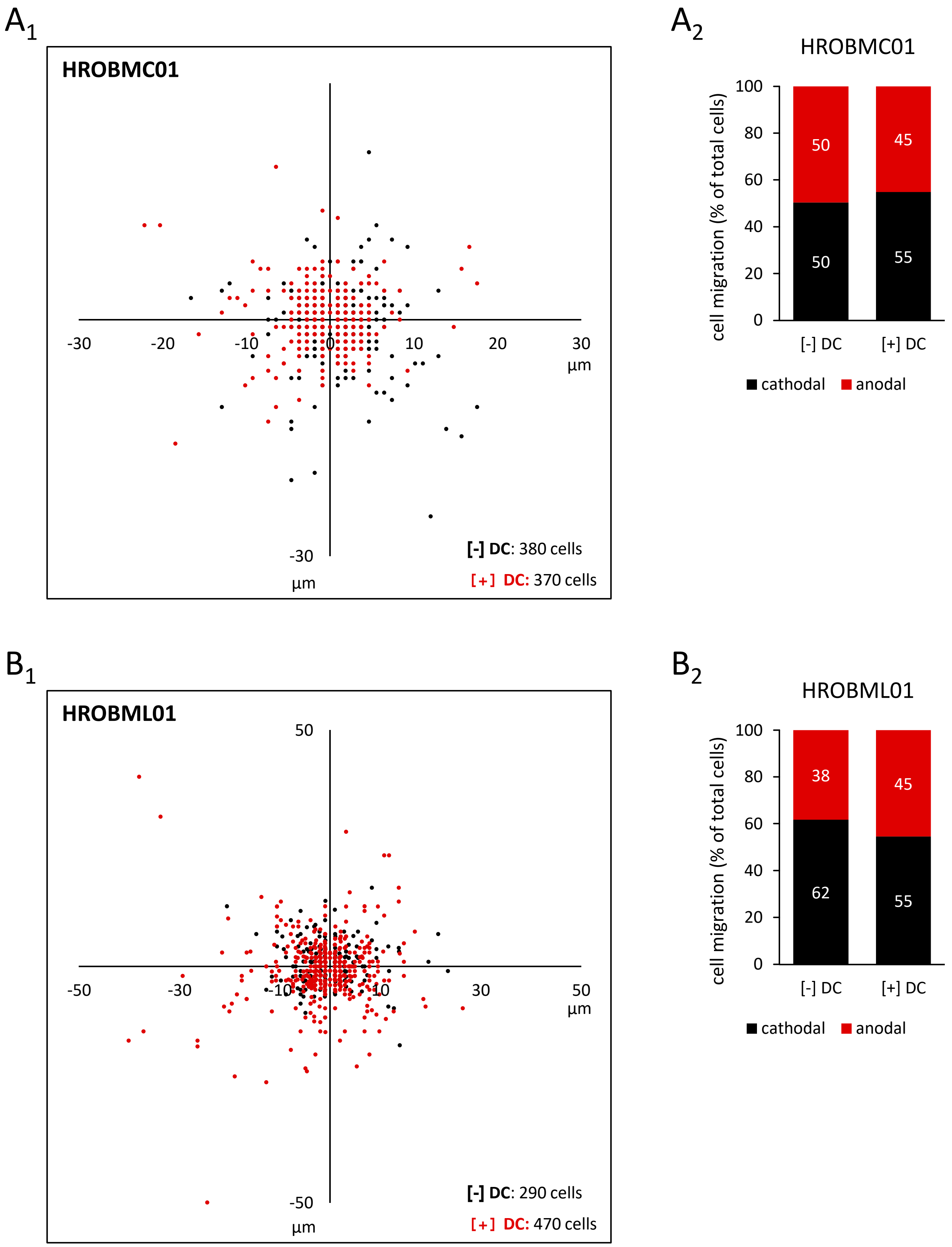

Supplementary Figure S5: Migration of [A_1] HROBMC01 and [B_1] HROBML01 metastases cells after 6 hours of DC electrical field stimulation is shown in red. Control cultures without DC are marked in black. Position of each cell is plotted in reference to the start of the experiment. [A_2] and [B_2] illustrate direction of galvanotactic migration ±DC.

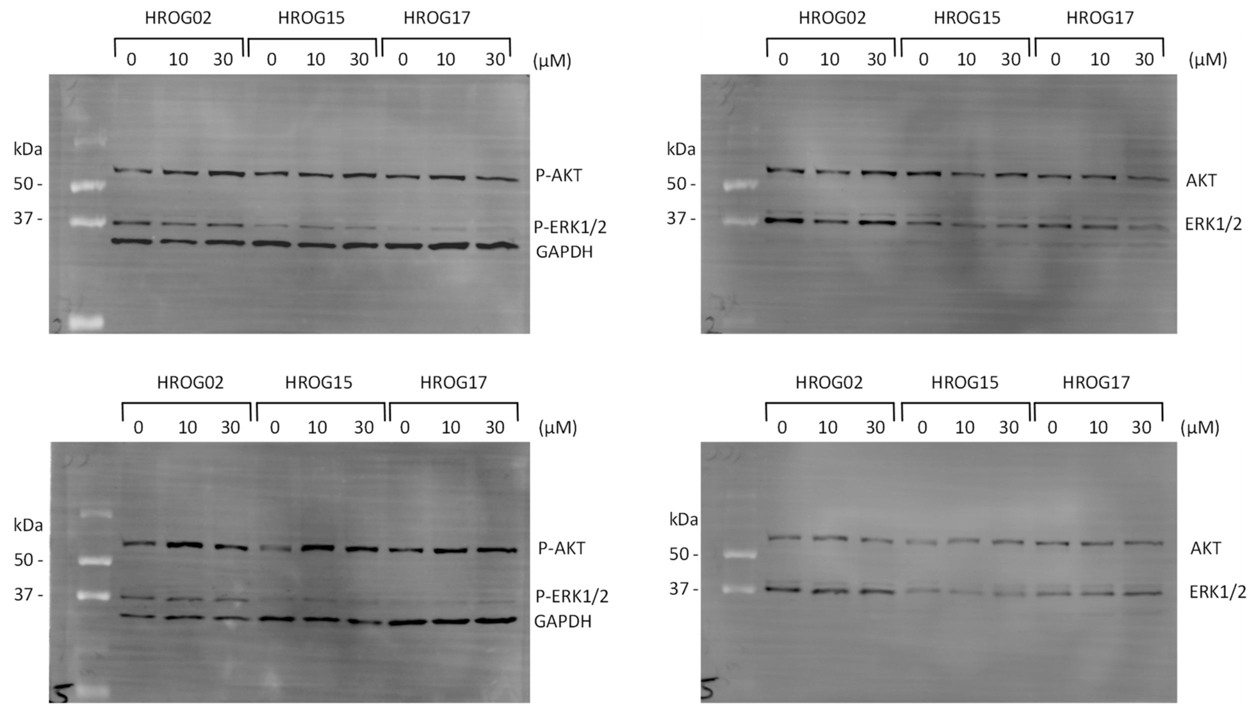

Supplementary Figure S6: Sample PVDF membranes of capivasertib effects on AKT and ERK1/2 activation. Precision Plus Protein Dual Color Standards (BIO-RAD) was used as molecular size marker. The blots were developed using LI-COR reagents for an Odyssey Infrared Imaging System and signal intensities of the investigated proteins were quantified by means of the Odyssey® software (Table S1). For further details see Materials and Methods section in the manuscript.

Table S1. Signal intensities from each protein band quantified in immunoblot analyses.

biological replicate		1			2			3			4			5			6		
Capivasertib (µM)		0	10	30	0	10	30	0	10	30	0	10	30	0	10	30	0	10	30
HROG02	P-Akt	3.74	0.52	14.28	4.38	6.66	11.28	6.00	7.07	13.82	6.33	4.59	11.09	4.63	13.38	8.12	10.59	6.83	16.11
	P-Erk1/2	4.99	1.18	2.76	6.14	3.52	4.42	6.05	6.06	6.32	3.92	2.60	4.01	3.98	3.96	3.08	5.10	1.82	3.65
	Akt	5.70	0.52	9.01	7.33	5.11	8.40	8.26	5.61	10.37	8.07	2.33	7.44	5.43	6.72	3.94	10.07	3.02	8.42
	Erk1/2	7.93	1.64	7.12	11.89	4.69	9.38	14.31	9.97	15.43	16.05	7.77	19.07	11.04	11.31	9.62	13.62	4.20	12.44
	GAPDH	10.56	0.62	13.99	15.04	9.26	14.36	12.12	7.42	17.82	10.42	4.32	11.21	4.75	8.01	4.85	15.51	3.87	9.18
HROG15	P-Akt	9.14	8.44	15.20	5.90	6.77	8.51	8.95	17.67	15.03	6.18	14.44	13.49	2.76	9.96	9.38	2.80	7.72	6.36
	P-Erk1/2	2.58	2.37	3.58	1.78	2.05	1.46	3.14	4.41	4.37	0.44	1.78	2.62	0.76	1.24	1.69	0.57	1.18	0.96
	Akt	10.15	3.66	8.59	6.11	2.68	4.55	11.25	10.13	10.76	9.69	9.77	10.34	2.99	3.71	5.24	3.59	3.63	3.74
	Erk1/2	4.23	0.58	3.95	2.56	0.12	2.47	9.88	8.72	11.12	9.99	8.76	9.83	3.54	2.54	4.74	3.48	2.48	1.98
	GAPDH	25.01	11.41	27.86	20.66	13.00	17.16	26.82	27.65	30.45	21.92	20.97	21.11	11.22	9.45	9.06	10.48	10.01	12.26
HROG17	P-Akt	6.79	9.53	8.09	5.30	9.15	6.57	5.66	8.95	6.69	8.42	7.13	10.31	6.34	10.35	10.58	4.57	6.44	7.84
	P-Erk1/2	0.38	0.92	1.91	0.47	1.33	1.57	1.16	1.82	1.97	0.40	0.93	1.89	0.90	0.96	0.94	1.35	1.12	0.90
	Akt	5.85	4.16	3.36	4.43	4.10	3.30	4.64	4.75	4.07	7.44	4.22	5.89	6.75	5.71	5.70	4.25	3.15	3.68
	Erk1/2	2.32	2.96	2.71	3.86	4.05	1.57	5.62	5.90	5.22	6.85	5.29	7.71	7.35	6.73	7.23	6.35	1.99	4.61
	GAPDH	18.73	14.77	14.47	18.80	24.70	18.67	16.66	17.69	20.30	14.03	11.98	20.96	17.37	17.84	23.74	15.12	10.39	15.19

Immunoblot analysis was performed employing LI-COR reagents for an Odyssey Infrared Imaging System and signal intensities of the investigated proteins (P-AKT, P-ERK1/2, AKT, ERK, and GAPDH) were quantified by means of the Odyssey® software. Table S1 presents data for 6 independent experiments with 6 biological replicates of all three cell lines (HROG02, HROG15 and HROG17). For further details see Materials and Methods section in the manuscript.
